# The Regulation of the NF-κB p65 and Nrf2/HO-1 Signaling Pathways by Fucoxanthin in Human THP-1 Monocyte Macrophages Under a Lipopolysaccharide-Induced Inflammation Model

**DOI:** 10.3390/foods14101746

**Published:** 2025-05-14

**Authors:** Linyi Zhang, Tong Li, Jingyi Liu, Jiyan Sun, Jinkun Niu, Dandan Ren, Yichao Ma, Yunhai He, Shu Liu, Qiukuan Wang

**Affiliations:** 1 College of Food Science and Engineering, Dalian Ocean University, Dalian 116023, China; zly060602@outlook.com (L.Z.);; 2 National R & D Branch Center for Seaweed Processing, Dalian 116023, China; 3 Key Laboratory of Aquatic Product Processing and Utilization of Liaonaing Province, Dalian 116023, China

**Keywords:** fucoxanthin, antioxidant, anti-inflammatory, THP-1

## Abstract

Fucoxanthin (Fx), a natural carotenoid predominantly found in brown algae and certain microalgae, has garnered significant attention in recent years for its potent antioxidant and anti-inflammatory properties. As inflammation and oxidative stress represent fundamental physiological responses that play pivotal roles in disease pathogenesis, their intricate interplay has become a focus of scientific investigation. This study employed an LPS-induced THP-1 cell inflammation model to elucidate the anti-inflammatory mechanisms of fucoxanthin and its interaction with oxidative stress pathways. Our findings demonstrate that fucoxanthin effectively suppresses the LPS-induced secretion of pro-inflammatory mediators, including IL-1β, IL-6, iNOS, COX-2, and TNF-α, in THP-1 cells. Mechanistically, this effect is achieved through the inhibition of IκB-α phosphorylation, thereby blocking the activation of the NF-κB p65 signaling pathway. Concurrently, fucoxanthin exhibits robust antioxidant activity, as evidenced by enhanced catalase (CAT) and superoxide dismutase (SOD) activities coupled with reduced malondialdehyde (MDA) production. Furthermore, fucoxanthin activates the Nrf2 signaling pathway, leading to upregulated heme oxygenase-1 (HO-1) expression and the consequent attenuation of reactive oxygen species (ROS) generation. These results collectively indicate that fucoxanthin exerts dual protective effects through anti-inflammatory action mediated by NF-κB pathway inhibition and antioxidant activity via Nrf2/HO-1 pathway activation. The observed crosstalk between these pathways suggests that fucoxanthin’s therapeutic potential stems from its ability to simultaneously modulate interconnected inflammatory and oxidative stress responses. Our study provides compelling evidence that fucoxanthin’s antioxidant and anti-inflammatory activities are functionally interrelated, with the Nrf2 signaling pathway serving as a critical node in this protective mechanism against LPS-induced cellular damage.

## 1. Introduction

Fucoxanthin (C_42_H_58_O_6_) is widely found in a variety of brown algae and some microalgae [1]. Due to the presence of isoprene and o-diene in the structure of fucoxanthin, it has a variety of bioactive effects, such as anti-inflammatory, anti-obesity, antioxidation, hypolipidemic activities, etc. [1].

Anti-inflammatory activity is the research hotspot of biological activity, and its mechanism mainly involves inhibiting the production of inflammatory factors, interfering with the signaling pathway of inflammatory response, and affecting the migration of leukocytes [2]. The common signaling pathways in inflammatory responses include mitogen-activated protein kinase (MAPK), Janus kinase/activator of signal transduction and transcription (JAK/STAT), nuclear factor-κ B (NF-κB), protein kinase B (AKT), and so on [3].

During the inflammatory response of cells, the NF-κB pathway is the most common pathway that regulates the inflammatory response and is critical for inflammation initiation [4,5]. Under stimulation, IκB kinase (IKK) protein is phosphorylated through a series of reactions, and then, IκB is phosphorylated and ubiquitinated. Polyubiquitinated IκB is recognized and degraded by 26S proteasome, and then, NF κ B dimers are released. These dimers are transferred to the nucleus in the cytoplasm to combine with DNA, which plays its role as transcription factor and induces the production of inflammatory factors [6].

As a marine carotenoid, fucoxanthin demonstrates remarkable free radical scavenging capacity under hypoxic conditions, which is attributable to the oxygen-containing functional groups in its molecular structure [7]. Furthermore, its unique allenic bond confers exceptional antioxidant properties [8]. The Nrf2 signaling pathway represents the primary endogenous antioxidant defense mechanism. Under basal conditions, Nrf2 remains sequestered in the cytoplasm through binding with its repressor protein Keap1. Upon oxidative stimulation, this Keap1-Nrf2 complex dissociates, enabling Nrf2 nuclear translocation where it initiates the transcription of antioxidant response element (ARE)-regulated genes, ultimately leading to the production of cytoprotective enzymes [9].

Researchers have found that oxidative stress in the body can lead to inflammation and that inflammation can also cause oxidative damage [10]. A high concentration of ROS can activate NF-κB, induce the synthesis of tumor necrosis factor-α, and then up-regulate the expression of inflammatory factors such as IL-6 and IL-1β [11,12]. The Nrf2/HO-1 signaling pathway can not only reduce the damage caused by free radicals, but it can also reduce the activity of nuclear transcription factor NF-κB and down-regulate the expression levels of inflammation-related genes, thus reducing the production of pro-inflammatory factors [13,14,15,16,17].

The human monocytic THP-1 cell line, originally derived from the peripheral blood of acute monocytic leukemia patients, serves as an established in vitro model for immunological studies. Following differentiation with appropriate stimuli (e.g., phorbol esters), THP-1 cells acquire macrophage-like phenotypes, making them particularly suitable for investigating inflammation and immune responses. This study utilized LPS to induce inflammation in THP-1 cells after differentiation. This aimed to investigate the anti-inflammatory activity and mechanism of fucoxanthin, as well as its impact on the interaction between the inflammatory response and oxidative stress. The results provide a potential theoretical foundation for future research and development on the efficacy of fucoxanthin.

## 2. Materials and Methods

### 2.1. Chemicals, Reagents, and Apparatus

Fucoxanthin was acquired from Shandong Jiejing Group Co., Ltd. (Rizhao, China) in a purity of over 90%. THP-1 cells were procured from the Shanghai Cell Bank of China Academy of Sciences, whereas MedChemExpress (Monmouth Junction, NJ, USA) provided Bay11-7082. RPMI1640 culture medium was obtained from GIBCO (New York, NY, USA), and the inflammatory factor elisa kit as well as the oxidase kit were purchased from Nanjing Jiancheng Bioengineering Institute (Nanjing, China). Q-pcr kit and primer were purchased from Hu’nan Accurate Biology Engineering Co., Ltd. (Changsha, China). MTT, SDS, and Tween20 were obtained from Beijing Solarbio Technology Co., Ltd. (Beijing, China). TRIS and glycine were acquired from Amresco (Solon, OH, USA). The PierceTM Rapid Gold BCA Protein Assay Kit and Albumen Marker were purchased from ThermoFisher Scientific Co., Ltd. (Waltham, MA, USA). The Real-time fluorescence quantitative PCR instrument (LightCycler96) was acquired from Roche (Basel, Switzerland). Western blot-related instruments were purchased from Beijing Liuyi Biological Technology Ltd. (Beijing, China).

### 2.2. Cell Culture of THP

The cells used in this experiment were derived from the Typical Culture Preservation Committee cell Bank of the Chinese Academy of Sciences (SCSP-567). THP-1 mononuclear macrophages were cultivated in RPMI complete medium, comprising 10% FBS, 100 U/mL of penicillin, and 100 μg/mL of streptomycin. The cells were cultured under specific conditions in a CO_2_ incubator at 37 °C with 5% CO_2_ and 90% humidity. The cell density was monitored and maintained in a range of 2 × 10^5^~4 × 10^5^ live cells/mL by observing under an inverted microscope every 24 h. The cells were passaged when the density reached 8 × 10^5^~1 × 10^6^ cells/mL, which corresponded to about 80% density. The experiment commenced when cells reached the logarithmic growth phase. THP-1 cells were differentiated into mononuclear macrophages upon addition of the inducer PMA. The addition of the inducer PMA Reagent to a final concentration of 0.1 µM transforms cellular THP-1 into monocyte macrophages for subsequent experiments.

### 2.3. MTT Assay Method

The cytotoxic effect of lipopolysaccharide (LPS) on THP-1 monocyte macrophages was assessed through the implementation of the MTT method [18]. Dilute the cell suspension, add inducer PMA reagent to make its final concentration 0.1 μM, inoculate 200 μL cells into a 96-well plate with a density of 3 × 10^4^ cells/well, leave a row of uninoculated cells (set as blank group 1), and put them into a CO_2_ incubator to make them stick to the wall. After 24 h of culture, carefully absorb the supernatant, wash the cells with PBS, discard the PBS, add 200 μL of fresh RPMI complete medium, and rest for 24 h. Dilute LPS with 1 × PBS. Carefully suck off the cell supernatant that has been resting for 24 h, gently add 190 μL of fresh RPMI complete medium, add 10 μL of PBS to blank group 2, and add 10 μL of LPS solution to other groups, so that the final concentrations are 1, 2, 4, 6, 8, and 10 μg/mL, and then put them into a CO_2_ incubator for 24 h. Set the sample group and the blank group with three parallel cells. Carefully suck off the cell supernatant, add 150 μL of prepared MTT solution (the final concentration is 0.5 mg/mL), and put it into a CO_2_ incubator for incubation for 3~4 h. Carefully suck off MTT solution of cell culture plate, add 150 μL DMSO, shake, and incubate at 37 °C for 10 min, then measure its absorbance at 490 nm [5], and calculate the cell survival rate. The formula is as follows: V = 100 × (AT − AN)/(AC − AN).

In the formula:

V—Cell survival rate;

AT—Absorption value of sample group at 490 nm;

AN—Absorption value of blank group 1 at 490 nm;

AC—Absorption value of blank group 2 at 490 nm.

The effects of different concentrations of LPS on the cytotoxicity of THP-1 mononuclear macrophages were assessed using the MTT assay. With the increase of fucoxanthin concentration, the cell viability of THP-1 showed a decreasing trend. When the concentration of LPS was 6 μg/mL, LPS began to have a damaging effect on cell viability. Therefore, LPS with a concentration of 4 μg/mL was selected for further experiments. Due to the high OD value during cytokine assay at a concentration of 4 μg/mL, it cannot be determined. Based on the literature and experiments, a concentration of 1 μg/mL was used for subsequent experiments.

### 2.4. CCK-8 Assay Method

As DMSO, the solvent utilized in fucoxanthin, has the potential to impact the MTT assay, the CCK-8 method was used to evaluate the harmful effects of fucoxanthin on THP-1 cells [5]. Dilute the cell suspension, add inducer PMA reagent to make its final concentration 0.1 μM, adjust the cell concentration to 3 × 10^4^ cells/well, and inoculate 200 μL of cells into a 96-well plate to induce THP-1 mononuclear leukemia cells to differentiate into mononuclear macrophages. After 24 h of culture, carefully discard the supernatant, wash the cells with PBS, discard PBS, add 200 μL of fresh RPMI complete medium, continue to culture for 24 h, discard the medium again, add 190 μL of fresh RPMI complete medium to each well, and add 10 μL of RPMI 1640 to the blank group. Add 10 μL of fucoxanthin solution (100× mother liquor with DMSO was diluted to the required concentration with RPMI1640) to the other groups, so that the final concentrations are 0.01, 0.1, 1, 5, 10, 20, and 30 μg/mL, and then culture it in in a CO_2_ incubator for 24 h, then add 10 μL of CCK-8 reagent to each hole, and continue culture them in CO_2_. Three parallel experiments were conducted in each group.

### 2.5. Griess Method for Measuring NO Content

Refer to Li’s method with some modifications [19]. Adjust the cell density of THP-1 cells to 1 × 10^6^ cells/mL, add the inducer PMA to make its final concentration 0.1 μM, quickly add the cell suspension into 96-well plates, and culture for 24 h, so that THP-1 mononuclear leukemia cells can be induced to differentiate into mononuclear macrophages. Discard the culture medium, wash the cells with PBS, discard PBS, add fresh RPMI complete culture medium 200 μL/well, and continue to culture for 24 h. Discard the culture medium, add 180 μL of fresh RPMI complete culture medium to each well, add 10 μL of RPMI 1640 to each well in blank group and LPS group, add fucoxanthin solution (100× mother liquor in DMSO was diluted to the required concentration with RPMI 1640) to the final concentrations of 0.1, 0.5, and 1 μg/mL, and then culture in carbon dioxide incubator for 2 h. An amount of 10 μL of PBS was added to the blank group, and 10 μL of LPS was added to the other wells. After 24 h of incubation in a carbon dioxide incubator, the cells were taken out, the cell supernatant was collected, and the concentration of NO in the cells was determined by the Griess method. Each group makes three parallel lines. Prepare solutions A and B as follows, reagent A: 6 mL of concentrated phosphoric acid (85%), 70 mL of deionized water, and 1.0 g of anhydrous p-aminobenzenesulfonic acid; reagent B: 0.1 g of N-(1-naphthyl)ethylenediamine hydrochloride is dissolved in deionized water. First, add 50 μL of standard solution or supernatant of cells to be detected to each well, add 50 μL of reagent A to each well, react in a carbon dioxide incubator at 37 °C for 10 min, then add 50 μL of reagent B to each well, and make it react in a carbon dioxide incubator at 37 °C for 10 min. Gently shake the 96-well plate several times, and after the reaction solution in each well is completely mixed, detect the OD value in each well at the wavelength of 540 nm.

### 2.6. Measurement of Inflammatory Factor Levels

The cell supernatant from 2.5 was collected, and the ELISA kit guidelines were followed to measure the levels of IL-1β, IL-6, iNOS, COX-2, and TNF-α. Three parallel experiments were performed for each group.

### 2.7. Measurement of Oxidase Activity and Oxidative Damage

Refer to Li’s method and make some modifications [5]. Adjust the concentration of cell suspension to make its cell density 1 × 10^6^ cells/mL, add inducer PMA to make its final concentration 0.1 μM, inoculate 1 mL of cells into a 24-well cell culture plate, and put it into a CO_2_ incubator to differentiate them into adherent cells. After 24 h of culture, the supernatant was carefully discarded, the cells were washed with PBS, PBS was discarded, 1 mL of fresh RPMI complete medium was added, and the cells were stably cultured in a carbon dioxide incubator for 24 h. Discard the culture medium, add 0.9 mL fresh RPMI complete culture medium to each well, add 50 μL RPMI1640 to each well of blank group and LPS group, add fucoxanthin solution (100× mother liquor with DMSO as solvent diluted to the required concentration with RPMI 1640) to make the final concentrations be 0.1, 0.5, and 1 μg/mL, and then culture in a carbon dioxide incubator for 2 h. An amount of 50 μL of PBS was added to the blank group, and 50 μL LPS was added to the other wells. After incubating for 24 h in a carbon dioxide incubator, the cells were taken out with a cell scraper, moved into a 1.5 mL centrifuge tube, and the supernatant was removed. After centrifugation, the supernatant was removed once or twice, and the required amount of extract was added, sealed with a sealing membrane, and the cells were crushed by ultrasonic wave (ice bath, power 100%, 40 min). The cells were treated according to the instructions of the kit, and the activities of SOD and CAT and the release of GSH and MDA were determined.

### 2.8. Intracellular Reactive Oxygen Species (ROS) Assay

The cells were treated as per protocol 2.7 and subsequently placed in a carbon dioxide incubator for 3 h after the addition of LPS. To prepare the DCFH-DA probe, it was diluted using serum-free culture solution at a 1:1000 ratio through the in situ loading probe method. The diluted probe was then spread evenly on the bottom of the cell culture plate. After leaving it to rest in the incubator for 30 min, the supernatant was removed, and the cells were washed twice using PBS [5]. Observations can be made directly through the use of a fluorescence enzyme-labeled instrument post-cell collection.

### 2.9. Gene Expression Measurement of NF-κB p65, Nrf2, and Their Associated Factors

Refer to Zhao’s method and make some modifications [20]. In addition to the fucoxanthin group, the inhibitor group (the addition of NF-κB inhibitor Bay11-7082 at a concentration of 10 μM) was also added. Following a 24 h incubation in a CO_2_ incubator, add the appropriate lysis solution Buffer RLS to cultured cells to lyse them [5]. Adjust the concentration of cell suspension, inoculate 2 mL cells into a 12-well plate to make the cell density 1 × 10^6^ cells/well, add inducer PMA to make the final concentration 0.1μM, and put it into a CO_2_ incubator to induce it to differentiate from mononuclear leukemia cells into mononuclear macrophages. After 24 h, carefully discard the supernatant, wash the cells with PBS, discard PBS, add 2 mL of fresh RPMI complete medium, and continue to culture in the cell incubator for 24 h. After discarding the culture medium, 1.8 mL of fresh RPMI complete culture medium was added to each well, 100 μL of RPMI 1640 was added to each well in the blank group and LPS group, and fucoxanthin solution (100× mother liquor in DMSO was diluted to the required concentration with RPMI 1640) was added to the other groups, so that the final concentrations were 0.1, 0.5, and 1 μg/mL. After culturing in a carbon dioxide incubator for 2 h, 100 μL of PBS was added to the blank group, and 100 μL of LPS was added to other wells (NF-κB inhibitor Bay11-7082 was added to the inhibitor group with a concentration of 10 μM, and DMSO was added to the other groups with the same volume). After incubation in a carbon dioxide incubator for 24 h, the cells were lysed with RNA extraction kit RLS lysate to collect cell samples. RNA was extracted using adsorption columns and then converted into cDNA using the reverse transcription reagent. This was then used to evaluate the expression of mRNA for NF-κB p65, IκB-α, IL-1β, IL-6, iNOS, COX-2, TNF-α, Nrf2, and HO-1 in each group of cells using fluorescence real-time quantitative PCR. The data were analyzed using the ΔΔ C (T) method.

Primer information of each index is as follows (Table 1):

### 2.10. Determination of Protein Expression of NF-κB, Nrf2, and Their Related Factors

The cells were treated according to 2.9, and the protein expressions of NF-κB p65, NF-κB p-p65, ikB-α, p-ikB-α, Nrf2, p-Nrf2, and HO-1 were determined by the WB method.

Li’s method was referred to, and some modifications were made [5]. Cells were harvested, and a suitable amount of reagents was added for the complete extraction of cellular proteins (protease inhibitors were added a few minutes prior to use) to obtain a total protein solution. To each sample’s total protein solution, 5× sampling buffer was added, mixed well, vortexed, centrifuged, and heated at 98 °C. Depending on the molecular weight of the proteins, two gels were created, one for separation and one for concentration during electrophoresis. Next, the PVDF membrane was transferred onto a separate membrane that had been immersed in an appropriate amount of 5% skimmed milk powder. The membrane was sealed and shaken for one hour on a decolorizing shaker. The primary antibody was diluted with diluent and the PVDF membrane was sealed. The sealed membrane was placed into a hybridization bag and incubated overnight at 4 °C. The membrane underwent five rounds of washing with TBST, each lasting five minutes at a time. Subsequently, the secondary antibody was diluted alongside 5% skimmed milk powder. Incubation with the secondary antibody took place for a duration of one hour. The process was repeated with TBST as appropriate, before the ECL mixture was administered onto the protein side of the membrane, triggering luminescence detection.

### 2.11. Statistical Analysis

The software GraphPad Prism 9.0 was used for data processing. The fucoxanthin concentration groups were compared with the blank control group and the positive control group, and the blank control group was compared with the positive control group. Significance analysis was performed by One-way ANOVA, and the result was expressed as mean ± standard deviation, with *p* < 0.05 indicating significant difference and *p* < 0.001 indicating extremely significant difference. Variable correlations were assessed using Pearson’s or Spearman’s tests, with data normality determined by Shapiro–Wilk tests.

## 3. Results and Discussion

### 3.1. Toxic Effect of Fucoxanthin on Human THP-1 Cells

**Figure 1:**
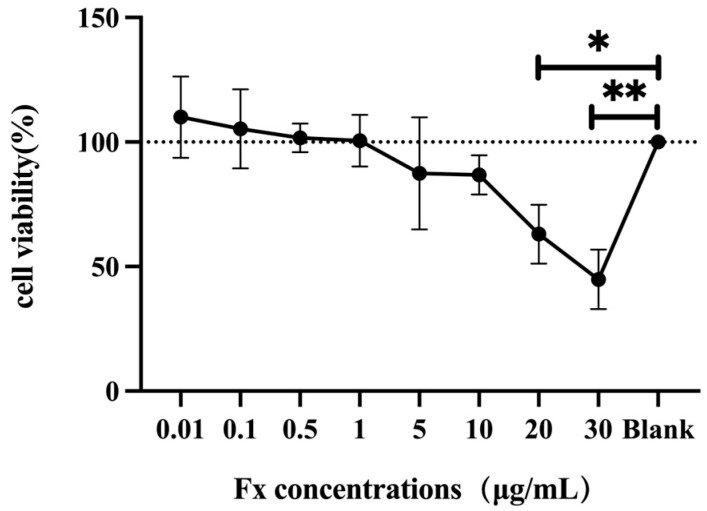
Determination of the effect of different concentrations of fucoxanthin on the viability of THP-1 cells by CCK-8 assay (the fucoxanthin concentration groups were compared with the blank control group for significance analysis, *: *p* < 0.05; **: *p* < 0.001).

Because cell reduction was already directly observable with the addition of high concentrations of fucoxanthin, we ultimately measured cell viability at 0.01–30 μg/mL of fucoxanthin addition. The effects of different concentrations of fucoxanthin on THP-1 cell viability were evaluated through the CCK-8 method. As shown in Figure 1, the viability of the THP-1 cell initially increased and subsequently decreased with the increase of fucoxanthin concentration. Fucoxanthin promoted cell proliferation when concentrations ranged from 0.01 to 1 μg/mL. When the concentration was higher than 5 μg/mL, fucoxanthin began to damage the cell viability. With the increase of the fucoxanthin concentration, the cell viability gradually decreased. When the concentration was 20 μg/mL, fucoxanthin significantly destroyed the cell viability (*p* < 0.05). When the concentration was 30 μg/mL, fucoxanthin reduced the cell activity to 44.87% (*p* < 0.001). Therefore, fucoxanthin with concentrations of 0.1, 0.5, and 1 μg/mL, which did not cause cell damage, was chosen for the follow-up study.

### 3.2. The Anti-Inflammatory Effect of Fucoxanthin on LPS-Induced Human THP-1 Cells

#### 3.2.1. Fucoxanthin Reduced LPS-Induced NO Production in THP-1 Cells

**Figure 2:**
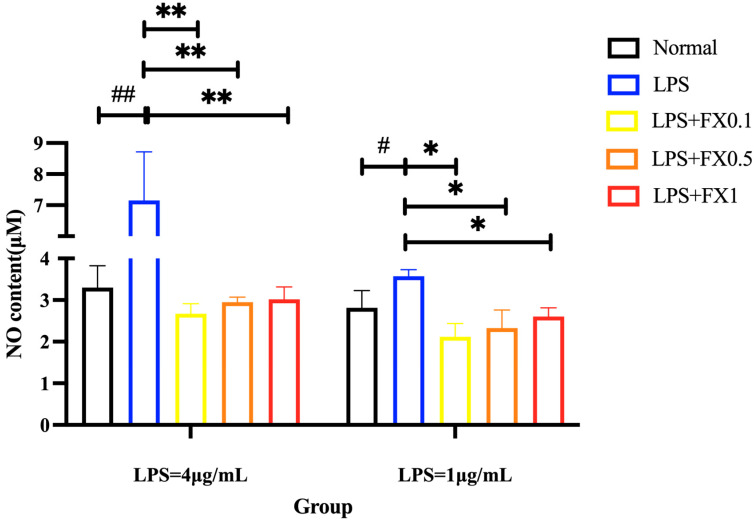
Effect of fucoxanthin on NO release from THP-1 cells induced by LPS (left: LPS = 4 μg/mL; right: LPS = 1 μg/mL) (significance analysis of normal group and LPS group, #: *p* < 0.05, ##: *p* < 0.001; significance analysis of the fucoxanthin concentration groups and the LPS group, *: *p* < 0.05, **: *p* < 0.001; LPS group was 1 μg/mL LPS; LPS + FX group (0.1, 0.5, 1) was 0.1, 0.5, and 1 μg/mL fucoxanthin concentrations co-administered with 1 μg/mL LPS).

At LPS concentrations of 1 and 4 μg/mL, the intracellular NO content in the model group was significantly higher than that in the blank group in Figure 2. Conversely, NO release content in the fucoxanthin group was significantly lower than that in the model group (*p* < 0.05). Under different concentrations of LPS treatment, fucoxanthin at concentrations of 0.1–1 μg/mL could reduce the high NO release caused by LPS to normal concentrations. The results showed that fucoxanthin can greatly inhibit the content of NO in THP-1 cells and ameliorate cellular inflammation.

#### 3.2.2. Fucoxanthin Decreased the Production of Inflammatory Factors Induced by LPS in THP-1 Cells

**Figure 3:**
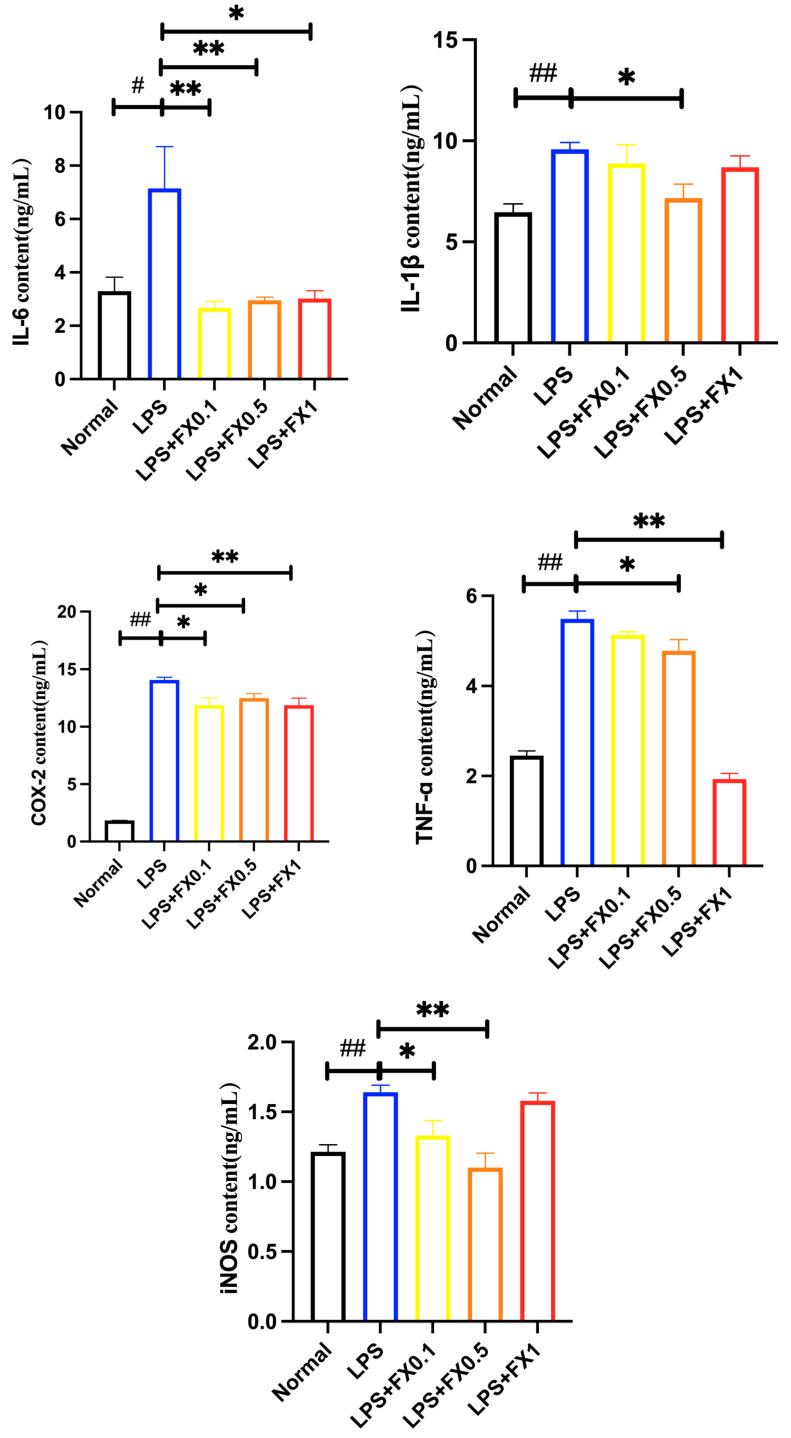
The effects of fucoxanthin on the release of IL-6, IL-1β, TNF-α, iNOS, and COX-2 from THP-1 cells induced by LPS (significance analysis of normal group and LPS group, #: *p* < 0.05, ##: *p* < 0.001; significance analysis of the fucoxanthin concentration groups and the LPS group, *: *p* < 0.05: **: *p* < 0.001; LPS group was 1 μg/mL LPS; fucoxanthin group (0.1, 0.5, 1) was 0.1, 0.5, and 1 μg/mL fucoxanthin concentrations co-administered with 1 μg/mL LPS).

As shown in Figure 3, when LPS was added, the concentrations of IL-1 β, IL-6, iNOS, COX-2, and TNF-α in the model group cells significantly increased (*p* < 0.05). Fucoxanthin effectively inhibited the production of IL-6 within the concentration range of 0.1–1 μg/mL. In addition, the lower the concentration of fucoxanthin, the stronger its inhibitory ability on IL-6, which was consistent with Liu’s previous research results [21]. After treatment with different concentrations of fucoxanthin, the content of IL-1 β in cells significantly decreased (*p* < 0.05). When the concentration of fucoxanthin was 0.5 μg/mL, the inhibitory effect on IL-1 β and iNOS in cells was the most significant, close to the blank group. After treatment with fucoxanthin, the content of TNF-α decreased significantly with the increase of fucoxanthin concentration (*p* < 0.05). When the concentration of fucoxanthin was 1 μg/mL, the inhibitory effect on TNF-α in cells was the best (*p* < 0.05). COX-2 is closely related to the NF-κ B signaling pathway, and it can participate in various disease response processes through NF-κ B, which is one of the important mechanisms leading to inflammatory diseases [22]. After treatment with fucoxanthin, the content of COX-2 in cells significantly decreased. When the concentration of fucoxanthin was 0.1 and 1 μg/mL, it had a highly significant inhibitory effect on COX-2 in cells. The results showed that a certain concentration of fucoxanthin can significantly inhibit the production of inflammatory factors such as IL-6, IL-1 β, iNOS, TNF-α, and COX-2 in THP-1 cells, effectively improving the inflammatory effect in THP-1 cells.

#### 3.2.3. Regulation of Fucoxanthin on NF-κB Signaling Pathway and Its Upstream and Downstream Genes in THP-1 Cell Inflammatory Model

**Figure 4:**
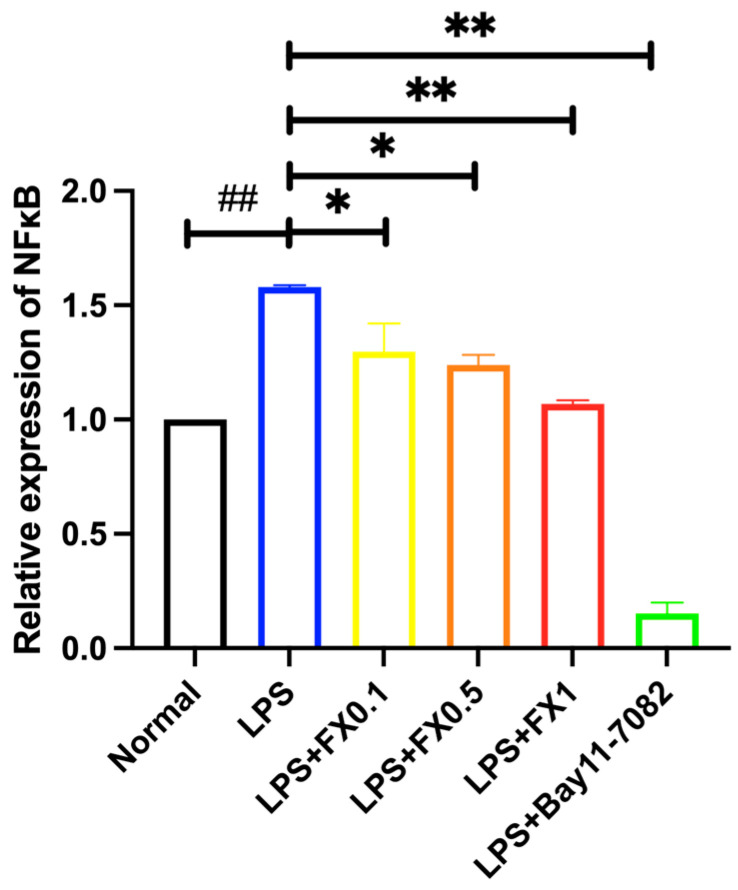
The effects of fucoxanthin and Bay11-7082 on the expression of NF-κB gene in THP-1 cells induced by LPS. (Significance analysis of normal group and LPS group, ##: *p* < 0.001; significance analysis of the fucoxanthin concentration groups and the LPS group, *: *p* < 0.05: **: *p* < 0.001; LPS group was 1 μg/mL LPS; fucoxanthin group (0.1, 0.5, 1) was 0.1, 0.5, and 1 μg/mL fucoxanthin concentration co-administered with 1 μg/mL LPS; LPS + Bay group Bay was 1 μg/mL LPS + NF-κB inhibitor Bay11-7082).

LPS treatment significantly upregulated NF-κB gene expression in THP-1 cells (*p* < 0.05), confirming the activation of the NF-κB signaling pathway. However, fucoxanthin co-treatment markedly suppressed (*p* < 0.001) this LPS-induced NF-κB overexpression in a dose-dependent manner. Notably, at a concentration of 1 μg/mL, fucoxanthin restored NF-κB expression to baseline levels comparable to those in untreated control cells. Meanwhile, it was also shown in Figure 4 that NF-κB inhibitor Bay11-7082, as an inhibitor, had a significant inhibitory effect on NF-κB, but the inhibitory effects of fucoxanthin at various concentrations on NF-κB were not as strong as NF-κB inhibitor Bay11-7082.

**Figure 5:**
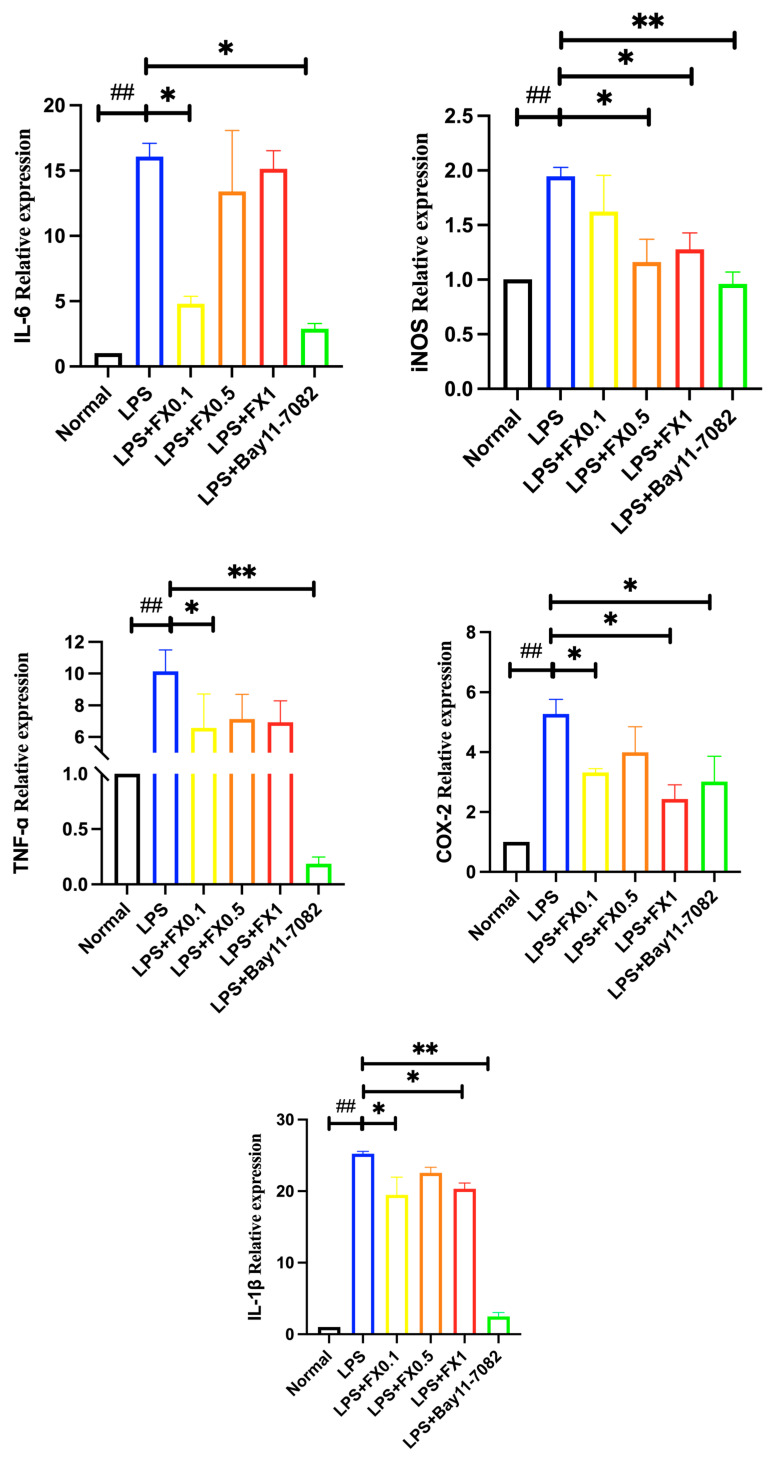
The effects of fucoxanthin and NF-κB inhibitor Bay11-7082 on IL-6, IL-1β, TNF-α, iNOS, and COX-2 gene expression in THP-1 cells induced by LPS (significance analysis of normal group and LPS group, ##: *p* < 0.001; significance analysis of the fucoxanthin concentration groups and the LPS group, *: *p* < 0.05: **: *p* < 0.001; LPS group was 1 μg/mL LPS; LPS + FX group (0.1, 0.5, 1) was 0.1, 0.5, and 1 μg/mL fucoxanthin concentrations co-administered with 1 μg/mL LPS; Group Bay was 1 μg/mL LPS + NF-κB inhibitor Bay11-7082).

As demonstrated in Figure 5, LPS treatment significantly upregulated the gene expression of pro-inflammatory mediators, including IL-1β, IL-6, iNOS, COX-2, and TNF-α, in THP-1 cells. This confirms that LPS robustly induces an inflammatory response. However, co-treatment with Bay 11-7082, a specific NF-κB inhibitor, effectively suppressed these inflammatory markers to near-normal levels, suggesting that NF-κB is a key regulatory pathway in this inflammatory cascade. Fucoxanthin also exhibited dose-independent inhibitory effects on these LPS-induced inflammatory genes at concentrations of 0.1–1 μg/mL (*p* > 0.05), though the suppression did not follow a clear concentration-dependent trend. Further investigation into the relationship between IL-1β, IL-6, iNOS, COX-2, and TNF-α and the NF-κB pathway revealed that Bay 11-7082 significantly downregulated their expression in the THP-1 inflammatory model (Figure 4), confirming the NF-κB-dependent regulation of these genes. Notably, Bay 11-7082 exhibited varying degrees of suppression across different inflammatory factors, implying that fucoxanthin’s anti-inflammatory mechanism may involve additional pathways beyond NF-κB inhibition.

#### 3.2.4. Inflammatory Pathway Protein Expression Was Inhibited by Fucoxanthin in THP-1 Cellular Inflammation Model

**Figure 6:**
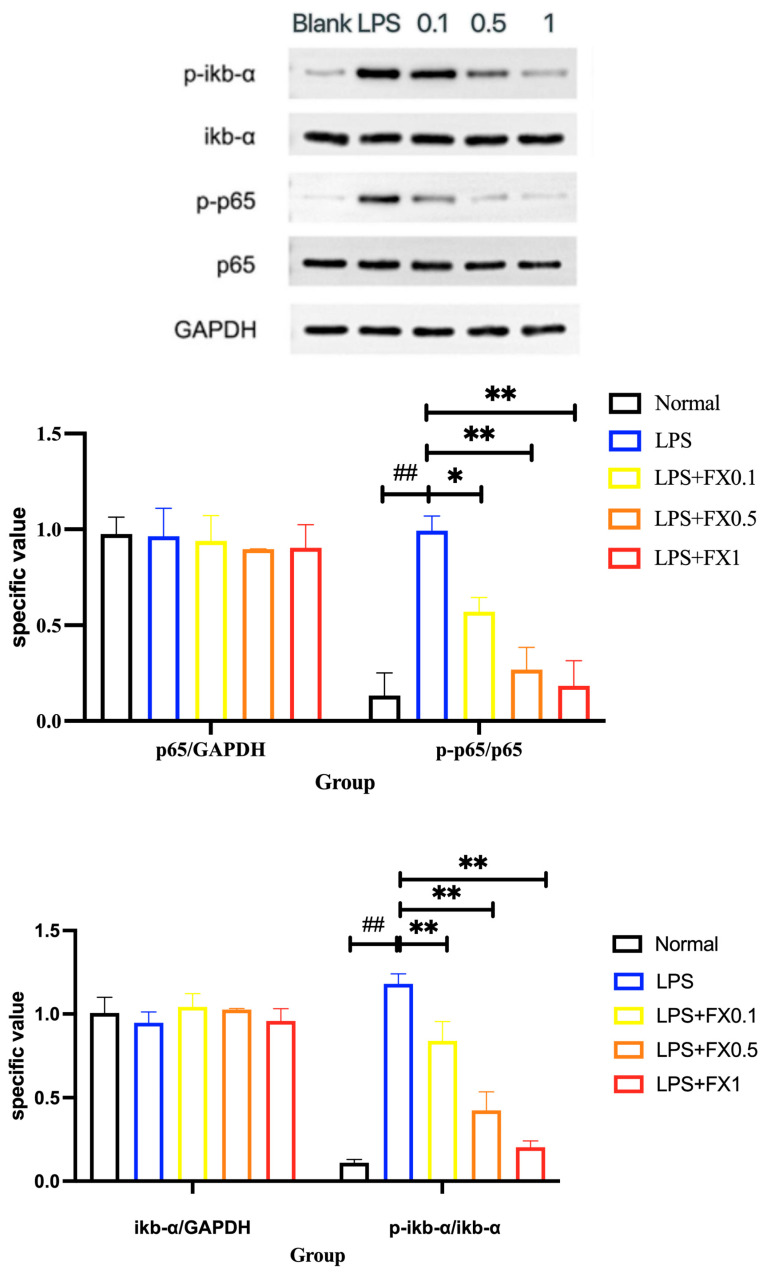
The effects of fucoxanthin on LPS-induced expression of NF-κB p65 and IκB-ɑ proteins in THP-1 cells (significance analysis of normal group and LPS group, ##: *p* < 0.001; significance analysis of the fucoxanthin concentration groups and the LPS group, *: *p* < 0.05: **: *p* < 0.001; LPS group was 1 μg/mL LPS; FX group (0.1, 0.5, 1) was 0.1, 0.5, and 1 μg/mL fucoxanthin concentrations co-administered with 1 μg/mL LPS).

To further investigate the relationship between fucoxanthin and NF-κB p65, we analyzed the expression of NF-κB p65-related proteins. As shown in Figure 6, LPS and fucoxanthin had no substantial effect on the overall protein of NF-κB p65 and its upstream factor, i-κB-α. The phosphorylation of NF-κB p65 and iκb-α in the blank state was low, and the addition of LPS significantly improved the phosphorylation of NF-κB p65 and iκb-α in THP cells (*p* < 0.05), indicating that the cell rapidly opens the NF-κB p65 signaling pathway to promote the phosphorylation of iκb-α and produce inflammation. The addition of fucoxanthin had an inhibitory effect, and the inhibitory ability was positively correlated with the concentration. These results suggest that fucoxanthin has a strong inhibitory effect on the inflammatory response of the NF-κB p65 pathway.

### 3.3. Protective Effect of Fucoxanthin on LPS-Induced Oxidative Reactions Human THP-1 Cells

#### 3.3.1. Effect of Fucoxanthin on Intracellular Antioxidant Enzyme Activity and GSH and MDA Production in THP-1 Cell Inflammatory Model

**Figure 7:**
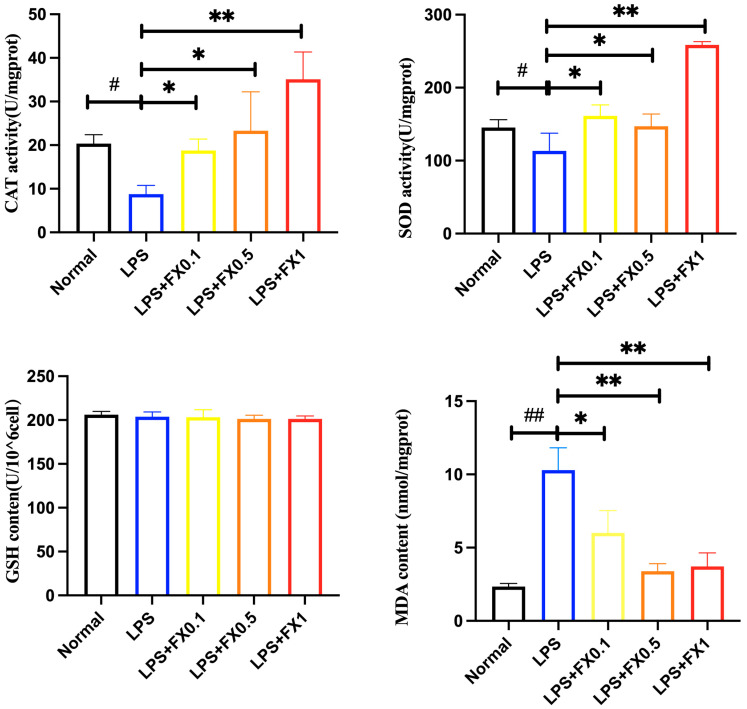
The effects of fucoxanthin on CAT, SOD, GSH, and MDA content in THP-1 cells induced by LPS (significance analysis of normal group and LPS group, #: *p* < 0.05, ##: *p* < 0.001; significance analysis of the fucoxanthin concentration groups and the LPS group, *: *p* < 0.05: **: *p* < 0.001; LPS group was 1 μg/mL LPS; LPS + FX group (0.1, 0.5, 1) was 0.1, 0.5, and 1 μg/mL fucoxanthin concentrations co-administered with 1 μg/mL LPS).

Oxidative stress also plays an important role in the inflammatory response; thus, the role of fucoxanthin in oxidative damage in the THP inflammatory model was investigated. From Figure 7, it can be concluded that LPS can significantly reduce the activity of CAT and SOD in THP-1 cells; thus, the inflammatory model can lead to oxidative stress in THP-1 cells. Adding 0.5–1 μg/mL of fucoxanthin can significantly increase the activity of CAT and SOD in cells (*p* < 0.05), with a concentration-dependent manner. After adding 0.5 μg/mL of fucoxanthin, both CAT and SOD can increase closely to the blank level. Therefore, fucoxanthin can significantly reduce the attenuation of CAT and SOD activities caused by the LPS inflammation model in THP-1 cells.

**Figure 8:**
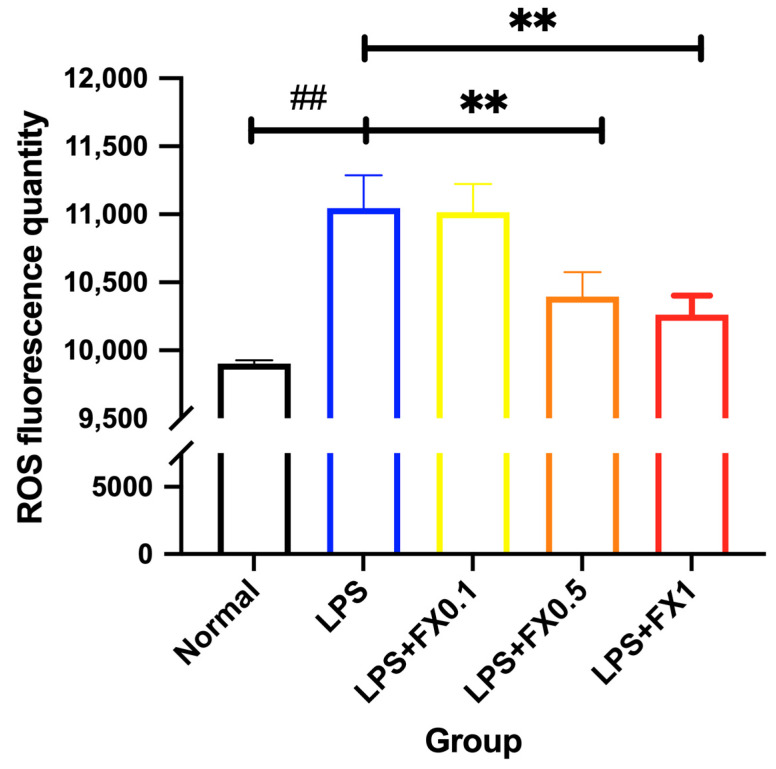
The effects of fucoxanthin on the fluorescence intensity of ROS in THP-1 cells induced by LPS (significance analysis of normal group and LPS group, ##: *p* < 0.001; significance analysis of the fucoxanthin concentration groups and the LPS group, **: *p* < 0.001; LPS group was 1 μg/mL LPS; LPS + FX group (0.1, 0.5, 1) was 0.1, 0.5, and 1 μg/mL fucoxanthin concentrations co-administered with 1 μg/mL LPS).

In Figure 7, the addition of fucoxanthin had no significant effect on GSH production. The results indicated that GSH in THP-1 cells may be insensitive to fucoxanthin at the experimental concentrations. The amount of MDA released is shown in Figure 8. LPS significantly increased MDA production in THP-1 cells, which demonstrated that the LPS-induced inflammation model in THP-1 cells can cause severe oxidative damage to the cells. The addition of fucoxanthin can significantly reduce the production of MDA in THP-1 cells and reduce it to blank levels. When 0.5 μg/mL of fucoxanthin was added, the inhibitory effect on MDA was most significant.

#### 3.3.2. Inhibition of Intracellular ROS Generation by Fucoxanthin in THP-1 Cell Inflammation Model

From Figure 8, it can be seen that the inflammatory reaction induced by LPS significantly increased the fluorescence of ROS. An amount of 0.1 μg/mL of fucoxanthin cannot significantly inhibit the increase of the fluorescence of ROS, but after adding fucoxanthin to 1 μg/mL, the fluorescence of ROS was obviously decreased (*p* < 0.05), which was close to the blank group. Whether the interaction between inflammation and oxidation by fucoxanthin was also related to this signaling axis remains to be studied.

#### 3.3.3. Fucoxanthin Activates the Nrf2 Signaling Pathway and Its Downstream Genes in a Model of THP-1 Cellular Inflammation

**Figure 9:**
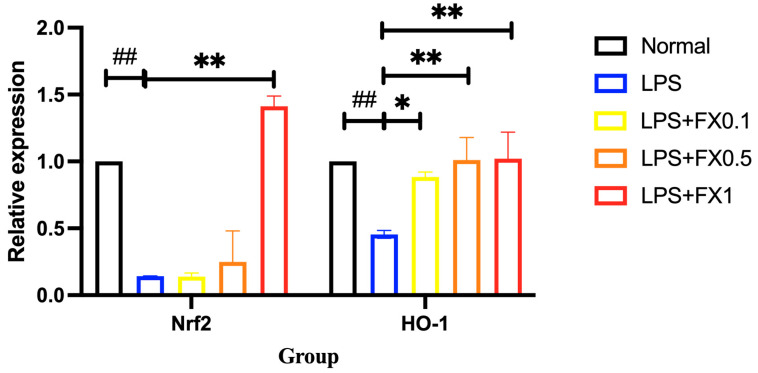
The effects of fucoxanthin on the relative expression of Nrf2 and HO-1 gene in THP-1 cells induced by LPS (significance analysis of normal group and LPS group, ##: *p* < 0.001; significance analysis of the fucoxanthin concentration groups and the LPS group, *: *p* < 0.05: **: *p* < 0.001; LPS group was 1 μg/mL LPS; LPS + FX group (0.1, 0.5, 1) was 0.1, 0.5, and 1 μg/mL fucoxanthin concentrations co-administered with 1 μg/mL LPS).

**Figure 10:**
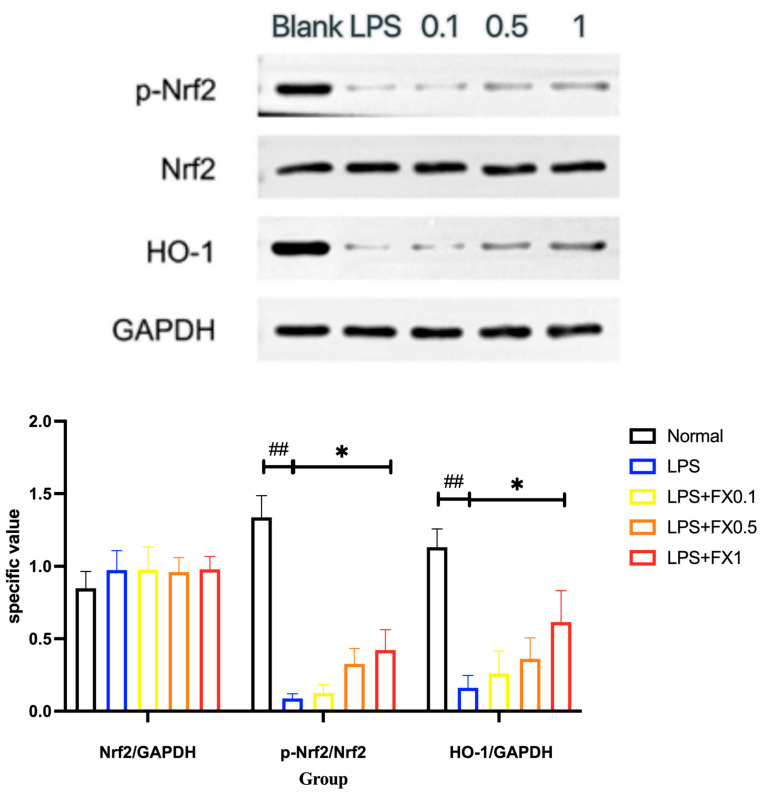
The effects of fucoxanthin on the expression of Nrf2 and HO-1 proteins in THP-1 cells induced by LPS (significance analysis of normal group and LPS group, ##: *p* < 0.001; significance analysis of the fucoxanthin concentration groups and the LPS group, *: *p* < 0.05; LPS group was 1 μg/mL LPS; LPS + FX group (0.1, 0.5, 1) was 0.1, 0.5, and 1 μg/mL fucoxanthin concentrations co-administered with 1 μg/mL LPS).

The Nrf2 signaling pathway is an important pathway for cellular oxidative stress. When stimulated by oxidation, Nrf2 and Keap1 dissociate into the nucleus, thus activating the expression of heme oxygenase -1(HO-1). Activated HO-1 can catalyze heme to decompose into biliverdin and release it, and then biliverdin is degraded into bilirubin by reductase, which has strong antioxidant capacity. From Figure 9, it can be seen that LPS can significantly inhibit the expression of the Nrf2 signaling pathway in THP-1 cells, while the addition of 0.1, 0.5, and 1 μg/mL of fucoxanthin can significantly increase the expression of the Nrf2 signaling pathway. The results of HO-1 in Figure 10 are also similar, and the results are similar to those obtained by Zhao [20] under the same conditions. It can be concluded that fucoxanthin can inhibit the LPS-induced ROS burst by activating the Nrf2 signaling pathway and downstream HO-1, which is concentration-dependent.

#### 3.3.4. Fucoxanthin Inhibits the Expression of the Nrf2 Signaling Pathway and Its Downstream Proteins in a THP-1 Cellular Inflammation Model

In the experiment, we concluded that the phosphorylation of Nrf2 in cells was significantly reduced when LPS was added, which may be due to the fact that THP-1 cells were macrophages induced by human mononuclear leukemia cells, and various signaling pathways in the cells remained active. After LPS was added, due to the inhibition of oxidative phosphorylation by LPS [23], Nrf2 was significantly reduced. Human Nrf2 protein contains six cysteine residues, cysteine (Cys183, Cys506), and other two key amino acid residues (Ser40, Tyr568) to regulate Nrf2 localization and target gene transcription through oxidative phosphorylation [24], so LPS can inhibit Nrf2 nuclear translocation. Fucoxanthin can significantly inhibit the production of this phenomenon. Under the action of fucoxanthin, Nrf2 translocates into the nucleus and binds to the upstream promoter of HO-1 to induce its expression. HO-1 is regulated by a variety of mechanisms, including mitogen-activated protein kinase, protein kinase C, reactive oxygen species, PI3K, and most importantly, the nuclear transcription factor Nrf2. As can be seen from Figure 10, LPS could significantly inhibit the phosphorylation of Nrf2 in THP-1 cells, thereby preventing the expression of the Nrf2 signaling pathway, while the addition of 0.1–1 μg/mL of fucoxanthin could significantly increase the protein expression of the Nrf2 signaling pathway. In addition, the protein expression trends of HO-1 and p-Nrf2 downstream were very similar, which further proves that fucoxanthin could improve the antioxidant capacity of cells by restoring the expression of the Nrf2 pathway. This result was consistent with the results obtained by Zhao, Pei, and Zhang et al. [20,25,26,27,28]. Therefore, the antioxidant regulation of fucoxanthin on THP-1 cells should be realized through the ROS/Nrf2/HO-1 signaling pathway [29].

## 4. Discussion and Conclusions

This study demonstrated the efficacy of fucoxanthin in treating inflammatory and oxidative damage caused by the LPS-induced inflammation model. Fucoxanthin shows antioxidant capacity. Physiological or pathological stressors induce reactive oxygen species (ROS) overproduction via mitochondrial electron leakage and NADPH oxidase activation, disrupting redox homeostasis and triggering oxidative stress cascades that compromise cellular integrity. Studies have shown that fucoxanthin can reduce the degree of lipid peroxidation in cells by targeting the removal of reactive oxygen species (ROS) on cells or mitochondrial membranes [30]. This effect can alleviate liver damage in mice models of nonalcoholic steatohepatitis (NASH) induced by choline deficiency and a high-fat diet, and it can also reduce mRNA expression levels of genes related to inflammation and inflammatory infiltration, inhibiting oxidative stress and inflammation in the liver [31]. Because of the close relationship between inflammation and oxidation, the inflammatory pathway and oxidation pathway activated by fucoxanthin have similar characteristics and targets. At the same time, studies have shown that fucoxanthin exerts anti-inflammatory and anticancer mechanism-related targets. Together, the proteins form disease-appropriate signaling pathways that play an anti-inflammatory and anticancer role.

By inhibiting IκB-α phosphorylation, fucoxanthin effectively blocked the activation of the NF-κB signaling pathway and reduced the release and expression of NO, IL-1β, IL-6, iNOS, COX-2, and TNF-α in THP-1 cells. However, the inhibitory effect of fucoxanthin on the expression of inflammatory cytokine genes may be achieved through multiple pathways. In this study, the inhibitory effect of fucoxanthin on TNF-α was only slightly reflected at the concentration of 1 μg/mL. Meanwhile, Sun [32] previously found in the study of an LPS-induced THP-1 cell model that the content of TNF-α peaked at 12 h within 0–24 h and decreased at 24 h, which was due to the autophagy of cells. The stimulation time of LPS in this experiment was 24 h; thus, the inhibitory effect of fucoxanthin on TNF-α production at low concentrations was not significant, which might be related to autophagy. Zhu [1] concluded that fucoxanthin has multiple mechanisms of anti-inflammatory action, such as NF-κB, TLR, PI3K, MAPK, AKT, and so on. Liu [33] noted in her article that fucoxanthin can significantly increase the activity of the AKT/PI3K signaling pathway. Hang [34] found that TNF-α in human glioblastoma A172 and mouse glioblastoma D1A cells was regulated by the PI3K-AKT signaling pathway through the addition of specific Akt inhibitors. In the resting state, the NF-κB protein binds stably to its inhibitory protein IκB in the cytoplasm. In diabetic hyperglycemia, the down-regulation of the PI3K/Akt pathway leads to the down-regulation of downstream protein IκB protein, the loss of inhibition of the NF-κB protein, and the activation of the NF-κB signaling pathway [35]. This indicates that TNF-ɑ may be jointly regulated by AKT and NF-κB. Lin [36] thought that IL-6 was related to JAK/STAT1 and JAK/STAT3. In this research, the expression of IL-6 in the 1 μg/mL fucoxanthin group was lower than that in the NF-κB inhibitor Bay11-7082 group, which was inconsistent with the rule of NF-κB in Figure 5, possibly because IL-6 was controlled by other pathways than NF-κB. This indirectly proves the possibility that fucoxanthin coordinates its anti-inflammatory effect through multiple inflammatory pathways.

High levels of fucoxanthin effectively restored the reduction of CAT and SOD activities in THP-1 cells that were induced by LPS, and they additionally decreased the elevation of MDA in THP-1 cells caused by LPS. However, GSH content was not significantly affected. Virginia thought that Nrf2 can promote the translocation of NF-κB p-50 to the nucleus by activating survival genes [37,38,39,40,41,42,43]. The activation of nuclear NF-κB is the final step of signal cascade activation induced by oxidative stress. Tang [44] found that Nrf2/HO-1 was involved in inhibiting the activation of nuclear NF-κB and that HO-1 was a key factor in the Nrf2-mediated suppression of NF-κB, which was supported by the fact that the NF-κB activity gradually decreased with the increase of Nrf2 in the experiments conducted in this article. Therefore, the anti-inflammatory and antioxidant interactions of fucoxanthin on THP-1 cells may be related to this association.

**Figure 11:**
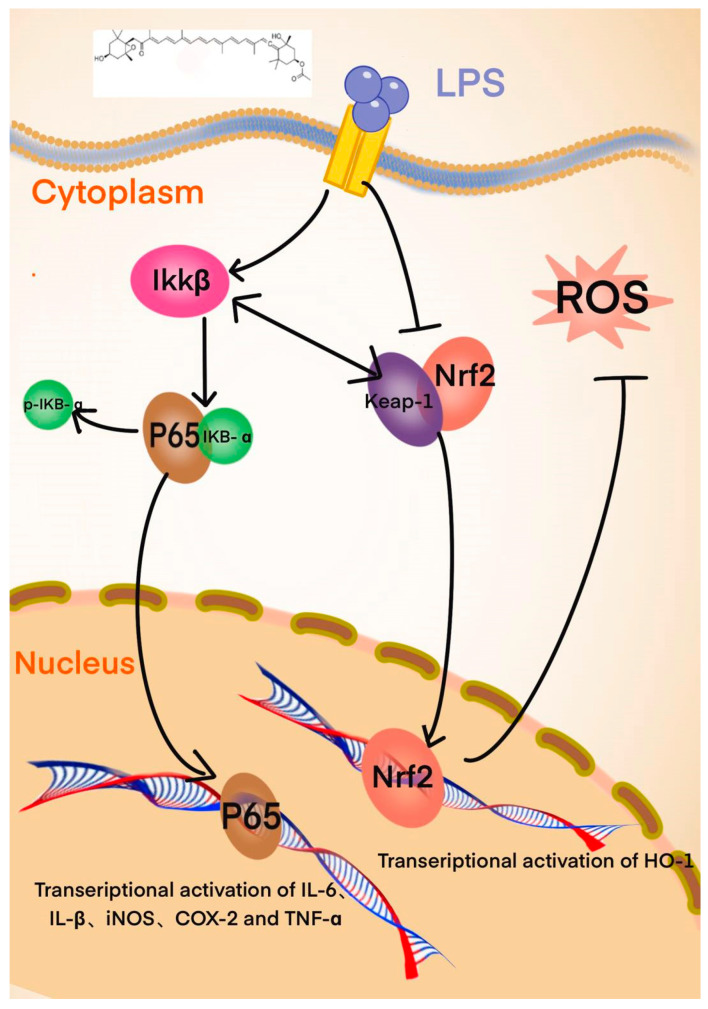
Nrf2 and NF-κ B signal path relationship diagram.

NF-κB is a key survival factor produced by lymphocytes [45]. In addition, NF-κB is sensitive to intracellular redox status and regulated by ROS and iNOS [11]. Braak believes that there is a strong correlation between the effectiveness of activating the Nrf2 pathway and the inhibition of NF-κB target gene expression [46,47,48], which is a direct relationship between oxidative and inflammatory pathways. The interaction between Nrf2 and NF-κB signaling is supported by the fact that the Nrf2 gene promoter region (NFE2L2) has several functional NF-κB binding sites [49]. Many antioxidants, such as low-dose quercetin, have been described in the literature to exert beneficial effects by activating Nrf2/ARE, promoting the expression of antioxidant enzymes (such as SOD), and activating cell survival pathways, as well as inhibiting the pro-apoptosis and pro-inflammatory genes regulated by NF-κB [50,51]. In addition, Nrf2 knockout mice are more sensitive to cytokine-induced inflammation [52]. Many studies have shown that, in the inactivated state, Keap1 binds to Nrf2 in the cytoplasm. When stimulated, the binding between Keap1 and Nrf2 becomes unstable, and Nrf2 enters the nucleus. The depletion of Keap1 induces the accumulation and stabilization of IKKβ, thus up-regulating the NF-κB reaction [53,54] (Figure 11). Moreover, the transfer of Nrf2 to the nucleus binds to antioxidant reactive elements (ARE) and p300; thus, the activation of Nrf2 leads to the depletion of p300, inhibiting p65 [55]. So, the competition between Nrf2 and NF-κB for p300 may also lead to the inhibition of the inflammatory response. Therefore, the addition of LPS inhibited the phosphorylation of Nrf2 in cells and released a large amount of reactive oxygen species, which may induce the activation of the NF-κB p65 signaling pathway, thereby promoting the phosphorylation of IκB-α and allowing NF-κB p65 to enter the nucleus, ultimately expressing factors related to inflammatory oxidation.

In conclusion, our study elucidates a dual mechanism whereby fucoxanthin counteracts LPS-induced inflammation through (1) the direct inhibition of NF-κB activation and subsequent inflammatory mediator production and (2) the activation of Nrf2/HO-1 signaling to mitigate oxidative stress. The reciprocal regulation between these pathways suggests that fucoxanthin’s therapeutic potential stems from its ability to simultaneously target multiple nodes in the inflammation–oxidation network. Future studies should further explore the molecular details of this crosstalk and investigate fucoxanthin’s broader bioactive properties at different molecular levels.

## Figures and Tables

**Table 1:** Primer design.

Gene Name	Sequence Content (5′ to 3′)
*GAPDH-F*	GCACCGTCAAGGCTGAGAAC
*GAPDH-R*	TGGTGAAGACGCCAGTGGA
*IL-6-F*	AAGCCAGAGCTGTGCAGATGAGTA
*IL-6-R*	TGTCCTGCAGCCACTGGTTC
*TNF-α-F*	TAAGAGGGAGAGAAGCAACTA
*TNF-α-R*	TCAGTATGTGAGAGGAAGAGA
*COX-2-F*	CCAGCACTTCACGCATCAG
*COX-2-R*	GCTGTCTAGCCAGAGTTTCACC
*iNOS-F*	CAGCAAGCAGCAGAATGAGTCC
*iNOS-R*	TGCATCCAGCTTGACCAGAGA
*IL-1β-F*	CCAGGGACAGGATATGGAGCA
*IL-1β-R*	TTCAACACGCAGGACAGGTACAG
*iκb-α-F*	CACTCCATCCTGAAGGCTACCA
*iκb-α-R*	AAGGGCAGTCCGGCCATTA
*NF-kB p65-F*	GACGCATTGCTGTGCCTTC
*NF-kB p65-R*	TTGATGGTGCTCAGGGATGAC
*Nrf2-F*	TGGGCCCATTGATGTTTCTG
*Nrf2-R*	TGCCACACTGGGACTTGTGTTTA
*HO-1-F*	TCTCTCTGGAAAGGAGGAAGGA
*HO-1-R*	AGGAACTGAGGATGCTGAAGG

## Data Availability

The original contributions presented in the study are included in the article. Further inquiries can be directed to the corresponding authors.

## Abbreviations

LPS: lipopolysaccharide, Fx: fucoxanthin, IL-6: interleukin-6, TNF-α: tumor necrosis factor-α, COX-2: cyclooxygenase-2, IL-1β: interleukin-1β, iNOS: induced nitric oxide synthase, NF-kB: nuclear factor κB, iκb-α: human nuclear factor κB inhibitor protein α, DMSO: dimethyl sulfoxide, Nrf2: nuclear factor-erythroid 2-related factor 2, HO-1: heme oxygenase-1, PMA: phorbol 12-myristate 13-acetate, Ikkβ: inhibitor of kappa B kinase, MAPK: mitogen-activated proteinkinase, ROS: reactive oxygen species.
